# The impact of universal mental health screening on stigma in primary schools

**DOI:** 10.1186/s13034-024-00854-5

**Published:** 2025-01-29

**Authors:** Annabel Songco, Deanna A. Francis, Emma A. McDermott, Chloe Y. S. Lim, Abigail Allsop, Joseph Croguennec, Gemma Sicouri, Andrew Mackinnon, Jennifer L. Hudson

**Affiliations:** 1 Black Dog Institute, University of New South Wales, Sydney, NSW Australia; 2 Faculty of Medicine and Health, University of New South Wales, Sydney, NSW Australia; 3 School of Psychology, University of New South Wales, Sydney, NSW Australia

**Keywords:** Universal screening, Child mental health, Primary school, Stigma, Anxiety, Depression

## Abstract

**Background:**

Primary school students struggling with mental health are less likely than high school students to access mental health care, due to barriers such as mental health stigma and low mental health literacy among children and parents. The near universal reach of schools offers a potential avenue to increase access to mental health care through early identification. The potential risks of this approach also need to be understood. This study monitored the impact of universal screening for mental health symptoms on stigma and mental health outcomes for primary school students.

**Methods:**

Across 6 primary schools, a cluster randomised controlled trial allocated schools to one of two conditions. Conditions varied based on the order and frequency of symptom and stigma questionnaires. A sample of 798 children (8 to 13 years; *Mage* = 10.29) completed assessments at baseline, 6-week, and 12-week follow-ups.

**Results:**

Significant time-by-group interaction effects were present, indicating differing changes in mental health stigma between groups. Follow-up analyses of subscales showed significant time-by-group interaction effects for concerns around self-stigma and secrecy, but not for public stigma. The frequency and presentation order of the questionnaires impacted on mental health stigma. Initially, children reporting on mental health symptoms before stigma, reported heightened stigma, but over time, those receiving more frequent presentations of the symptom check experienced an overall stigma reduction, contrasting with an increase in the comparison group.

**Conclusion:**

The frequency and presentation order of mental health symptom assessments impact children’s reports of mental health stigma, underscoring the importance of screening context. Potential screening harms, such as exacerbating self-stigma and secrecy, warrant consideration. Addressing stigma-related barriers is crucial for enhancing mental health care access for children in schools.

*Trial registration* Australian and New Zealand Clinical Trials Registry (ACTRN12622001114730) https://www.anzctr.org.au/Trial/Registration/TrialReview.aspx?id=384472 Date of trial registration: 12th August 2022.

**Supplementary Information:**

The online version contains supplementary material available at 10.1186/s13034-024-00854-5.

## Introduction

Anxiety and depression are among the most common mental health disorders in Australian children [1]. Whilst research shows no evidence of an increase in anxiety and depressive disorders in children [, 2], there has been a substantial increase in reported symptoms of anxiety and depression [3, 4]. A recent meta-analysis conducted during the COVID-19 pandemic showed a rise in global prevalence rates of anxiety and depression symptoms in children and adolescents (aged 18 years old or less), indicating that 1 in 4 experienced clinically elevated depression symptoms, and 1 in 5 experienced clinically elevated anxiety symptoms [3]. A study conducted in Australia during the initial stages of the COVID-19 pandemic, found that 1 in 5 children and adolescents (aged 4 to 17 years old) experienced considerable symptoms of anxiety and depression characteristic of a mental health disorder [4]. The high prevalence of mental health problems in children is a significant public health concern given the health, social, and economic burden [5, 6]. Thus, urgent action is needed to address the mental health of young people, in particular primary school aged children (5 to 12 years old), when the onset of mental health problems can begin.

Anxiety and depressive disorders in childhood can have a negative impact on a child’s educational, social, and emotional development, and when left untreated, can lead to increased risk for mental disorders into adolescence and adulthood [7]. Alarmingly, research shows that 50% of mental disorders emerge before the age of 14 and 75% by 24 years of age [8]. Indeed, anxiety and depression are not often identified until adolescence, when comorbidity is greater, and symptoms are harder to treat. However, given the early onset of anxiety and depressive symptoms, childhood provides a critical window of opportunity for implementing early intervention and prevention programs to reduce the growing global burden of disease.

Currently, the identification of mental health disorders in children is low, and even after identification, few children access the psychological care needed for support. Barriers such as long waitlists, a shortage in mental health professionals, poor implementation of treatment, and low mental health literacy around knowledge and beliefs around mental disorders, has led to limited access to mental health care and help-seeking behaviours [9]. In Australia, only 20% of children aged 4 to 11 years old with mental disorders receive mental health support from a psychologist [1]. Furthermore, primary school children are less likely than high school children to receive mental health care, with 40% of Australian youth not receiving any care in the school or health system [10]. Addressing the significant gap in the delivery of evidence-based services and support for primary school children during this important period of development is a priority.

One of the most common barriers to accessing support for mental health services and help-seeking behaviours in young people is stigma [11–13]. Mental health stigma can manifest in different ways and typically involves negative attitudes or beliefs surrounding mental health problems and is associated with discrimination or embarrassment [14]. Stigma can be explicit (conscious, controllable negative attitudes and beliefs) or implicit (subconscious, automatic negative attitudes and beliefs) [15]. Further, the multidimensional nature of stigma means the construct can be conceptualised in many ways, with types of stigmas including self-stigma, public stigma, personal rejection, and secrecy surrounding disclosure of mental health problems [16]. A deeper understanding of the different types of stigmas is crucial when determining the impact of mental health stigma faced by young people [17]. Evidence suggests that children and adolescents with mental health problems experience stigmatisation due to concerns that a mental disorder label may reduce life or social opportunities, and self-esteem [18]. Mental health stigma can develop early in childhood, with children associating mental disorders with unpredictability and violence or personal failure and weakness [19]. Understanding concerns surrounding mental health stigma and improving mental health literacy for children and their families may facilitate help-seeking behaviours and use of mental health services.

School-based interventions that aim to promote wellbeing and prevent mental health problems are an important strategy for increasing access to psychological support, reducing stigma, improving mental health literacy, and increasing help-seeking behaviours in children. Delivering mental health interventions through schools can be advantageous as schools provide a familiar setting in which to normalise and educate children about mental health, increase accessibility in reaching a large number of children from varied backgrounds, and school staff are often the first port of call for identifying mental health problems and offering support to children in need of help [20–22]. However, it is widely debated in the literature whether school-based mental health interventions may potentially cause harm due to privacy challenges, risks associated with the identification of children who need attention [23–26], and stigma-related concerns such as accessing mental health support in proximity to peers and teachers [27, 28]. A recent systematic review found that school-based mental health interventions were moderately effective in improving mental health literacy and reducing mental health stigma, although it was not clear if effects were sustained long-term with a lack of studies including follow-up assessments [29]. Empirical evidence on stigma and school-based services is mixed, as some studies suggest reductions over time in mental health stigma [30–35] and stigmatising attitudes and beliefs [, 32–35]. Whilst other studies in young people have shown stigma gradually worsened over time [36, 37] or showed no improvements [38–42]. Given the increasing pressure for school-based services to expand access to mental health care for children, it is critical to understand how stigma-related concerns might compromise the effectiveness of interventions and how these challenges can be overcome.

The optimal design for delivering school-based interventions has been largely contested in the literature, with mixed results for the effectiveness of universal or targeted intervention approaches for early identification of mental health problems in children [, 43]. Whilst targeted interventions are aimed at selecting children at-risk for developing a mental disorder (e.g., those with high absentees, poor academic performance, family concerns, self-selected, or selected by a parent or teacher), universal intervention approaches are delivered to whole populations (e.g., whole classes, schools or regions) offering a wider reach to diverse groups of children [44]. Universal screening approaches can have the additional benefits of intervening early to prevent the onset of mental health problems in children by addressing community-wide factors associated with improving positive mental health, help-seeking behaviours, and raising awareness in mental health literacy [,, 45]. Furthermore, universal intervention and universal screening approaches may be favourable as they do not require singling out children at-risk of mental disorders in front of their peers, which can potentially lead to further stigma and decrease program engagement [43, 46].

Despite schools providing a good opportunity for early interventions and screening, the undertaking of mental health screening in schools is low. One study that investigated the barriers, feasibility, and acceptability of school-based mental health approaches in Australia, found a low overall uptake of mental health screening (15%) in Government, Catholic, and Independent schools [47]. Further, 16% of students identified as at-risk were not followed-up or provided with resources to support their mental health and approximately 66% of school psychologists reported that they could not cope with increased demands that would result from screening. These data highlight the critical constraint facing most schools that have limited mental health resources, capacity, and staff to support children struggling with their mental health. There have been few studies to date that have evaluated early identification methods of screening in Australian schools and assessed potential harms associated with school-based interventions [47–52]. A recent study by Braund and colleagues [48] found no potential mental health related harms associated with the universal screening of anxiety and depression administered in Australian high schools. In this cluster randomized controlled study, schools were randomised to receive either an intensive screening procedure or a light touch screening procedure to examine the effectiveness of a digital mental health service for improving adolescents’ help-seeking behaviours. The study found no risk of harm relating to anxiety and depressive symptoms, distress, or deterioration in help-seeking intentions or mental health stigma. Whilst this is promising, most studies have been conducted in adolescents [48–51] with no studies to date that have examined potential mental health harms associated with universal screening in Australian primary school children. One study conducted in the UK [53] surveyed parents on their views on the acceptability of mental health screening in primary schools. Whilst majority of parents (82%) thought that mental health screening in primary schools would be helpful, some parents (13%) voiced concerns around the perceived harms of screening such as inaccurate identification, stigmatisation, and low availability of follow-up care. A better understanding of the barriers associated with delivering universal mental health screening in primary schools and further insights into stigma-related concerns around school-based universal screening, in primary school aged children, is needed.

The context, study design, and implementation of mental health screening checks in schools are important factors to consider when administering universal screening in terms of potential benefits and harms. For instance, the order and frequency in which questionnaires are presented to students may play a role in influencing responses and outcome measures. Research on context effects [54] suggests that the order of questions can shape responses by affecting how participants interpret and process subsequent questions. Preceding questions can influence responses to subsequent questions, affect their inferences about subsequent questions [55], and activate general norms applied to other issues [56]. Further, the order in which questionnaires are presented has been shown to impact response patterns [], with potential cognitive and affective priming effects [57, 58]. Given the gap in the literature in understanding the potential mental health related harms associated with universal screening approaches in primary schools, careful consideration should be given to the context, presentation order, and frequency of mental health and stigma questionnaires in universal screening in schools.

The present study examined the impact of assessing mental health symptoms on mental health stigma in primary school children. In particular, we were interested in understanding how the presentation order and frequency of the symptom checks may influence stigma, with important implications for the way universal checks are administered in school settings. The primary aim of the study was to monitor whether asking children about their mental health had an impact on stigma in children over time. The study design involved two conditions (i.e., monitor A group vs. monitor B group), which varied based on the order and frequency of symptom and stigma questionnaires. The monitor A group completed the symptom measure first, followed by the stigma assessment at each timepoint, whilst the monitor B group completed the stigma assessment at all timepoints, and completed the symptom measure *after* the stigma assessment only at baseline and at 12-week follow-up. The secondary aim of the study was to assess the monitoring of anxiety and depression symptoms in children. Consistent with research demonstrating that increasing mental health knowledge can reduce stigma [29] and together with previous literature on context and ordering of questionnaires, we hypothesised that exposing students to answer questions about their mental health symptoms before completing the stigma questionnaire (monitor A group), will be more effective in reducing stigma than if the stigma questionnaire were administered first (monitor B group). By presenting the symptom checks before the stigma questionnaire and at more frequent timepoints, we aim to leverage order effects and repetition that may increase mental health awareness and potentially lead to reduced stigma over time. In line with previous literature on anxiety and depression trajectories in children [59, 60], we also hypothesised that anxiety and depression symptoms would decrease over time in both groups due to potential increased awareness and exposure to mental health questionnaires. This study has important implications for administering universal checks in schools and better understanding the impact of mental health screening questions on stigma.

## Method

### Participants

Participants in the study were 798 primary school children aged 8 to 13 (*Mean* age = 10.29, *SD* = 1.23) across Australia. A small sample of parents/carers (n = 30) also participated. Government and Independent primary schools were eligible to participate if they had the capacity to provide children with a device (e.g., laptop, desktop, or tablet) to complete the study. Primary school children in grades 3 to 6 were eligible to participate, whilst children in grades Kindergarten (K) to 2 were considered too young to provide self-report and independently answer mental health questions, due to the required literacy skills. Parents/carers were eligible if they had a child in grades K to 6 from a participating school, access to a device to complete the study, and the capacity to consent and complete questionnaires. However, parent/carer data were excluded from the analyses due to low response rates and retention across all three timepoints (n = 20). Therefore, in the present study, only child data and measures are reported and analysed.

**Fig. 1:**
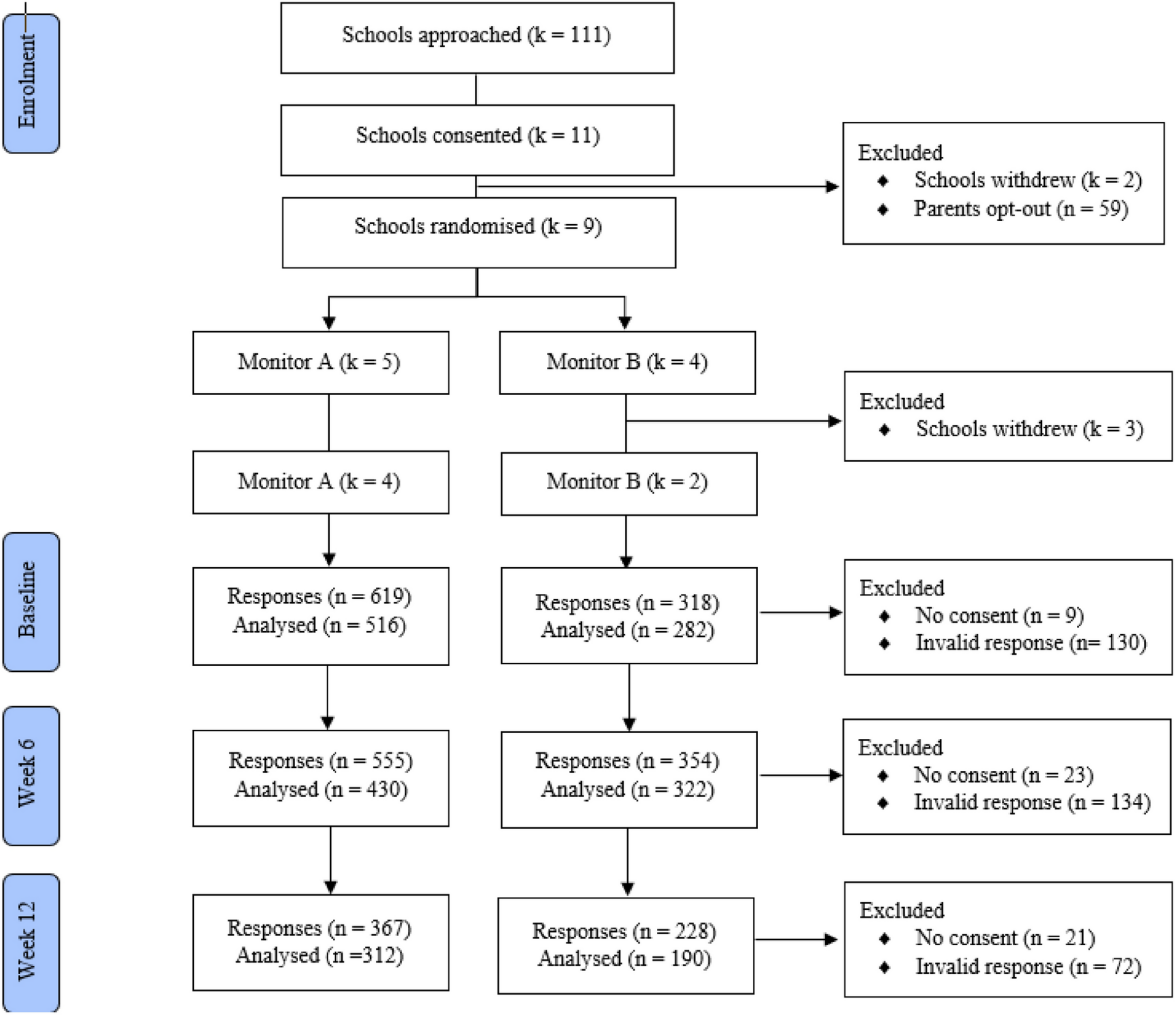
Consort diagram of participants through the study. Reasons for school withdrawals included scheduling and administrative issues (*k* = 2), concerns regarding the mental health content (*k* = 2), and concerns regarding the opt-out process and confidentiality (*k* = 1). Parent opt-out refers to the parent choosing that their child would not participate in the school-based study. Invalid responses included incomplete responses, duplicate responses, and responses completed at the incorrect timepoint

The final sample comprised of six eligible participating schools, which included co-education (*n* = 5) and single-sex (*n* = 1) schools from both metropolitan (*n* = 4) and rural (*n* = 2) regions of Australia, and Government (*n* = 1) and Independent schools (*n* = 5), with class sizes ranging from 15 to 35 students. Demographic factors of participating children can be found in Table 1. Figure 1 presents the CONSORT flow of the trial.

**Table 1:** Demographic characteristics of participants at Baseline

Variable	Monitor A		Monitor B		Total		Test^†^
	n	%	n	%	n	%	
Age							^*χ*2^(1) = 0.36, *p* = 0.5501
Mean *(SD)*	10.21 *(1.24)*		10.39 *(1.21)*		10.29 *(1.23)*		
Range (years)	8–13		8–13		8–13		
Grade							^*χ*2^(1) = 2.33, *p* = 0.1269
3	97	23.4	46	16.3	143	20.5	
4	87	21.0	51	18.1	138	19.8	
5	103	24.9	76	27.0	179	25.7	
6	127	30.7	109	38.7	236	33.9	
Gender							^*χ*2^(4) = 1.77, *p* = 0.7771
Female	279	57.3	124	44.0	403	52.4	
Male	185	38.0	141	50.0	326	42.4	
Don’t want to say	13	2.7	10	3.6	23	3.0	
Not sure	9	1.9	7	2.5	16	2.1	
Other	1	0.2	0	0	1	0.1	
Ethnicity							^*χ*2^(8) = 9.40, *p* = 0.3098
White	159	38.7	136	48.4	295	42.6	
European	19	4.6	6	2.1	25	3.6	
Asian	51	12.4	37	13.2	88	12.7	
North/Sub-Saharan African/Middle East	23	5.6	3	1.1	26	3.8	
Oceanian	10	2.4	5	1.8	15	2.2	
South/Central American	6	1.5	3	1.1	9	1.3	
Other/mixed	87	21.2	49	17.4	136	19.7	
Not sure	46	11.2	30	10.7	76	11.0	
Don’t want to say	10	2.4	12	4.3	22	3.2	
Aboriginal and/or Torres Strait Islander							^*χ*2^(3) = 9.61, *p* = 0.0222
No	300	64.4	211	74.8	511	68.3	
Not sure	128	27.5	45	16.0	173	23.1	
Yes	15	3.2	7	2.5	22	2.9	
Don’t want to say	23	4.9	19	6.7	42	5.6	

^†^ Likelihood ratio tests of between-group differences based on comparison of mixed models appropriate to level of measurement with and without group factor but including random school intercept

### Study design

This study was a cluster randomised controlled trial (cRCT). Participating schools were randomly allocated to either one of two conditions; a monitor A group or monitor B group, and completed assessments at three timepoints: baseline, 6-week follow-up, and 12-week follow-up. The 6-week and 12-week follow-up questionnaires were scheduled based on the first timepoint when students completed the baseline assessments. Two weeks prior to the scheduled follow-ups, schools were contacted to ensure the dates were still suitable and if necessary, were moved to the nearest convenient date for the school.

The study was preregistered with the Australian and New Zealand Clinical Trials Register (ACTRN12622001114730) on the 12th August 2022. The trial received ethics approval from the University of New South Wales Human Research Ethics Committee (HC Reference number: HC220332). Approval was also received from the New South Wales Department of Education (SERAP 2022075) to conduct research in Government schools.

**Fig. 2:**
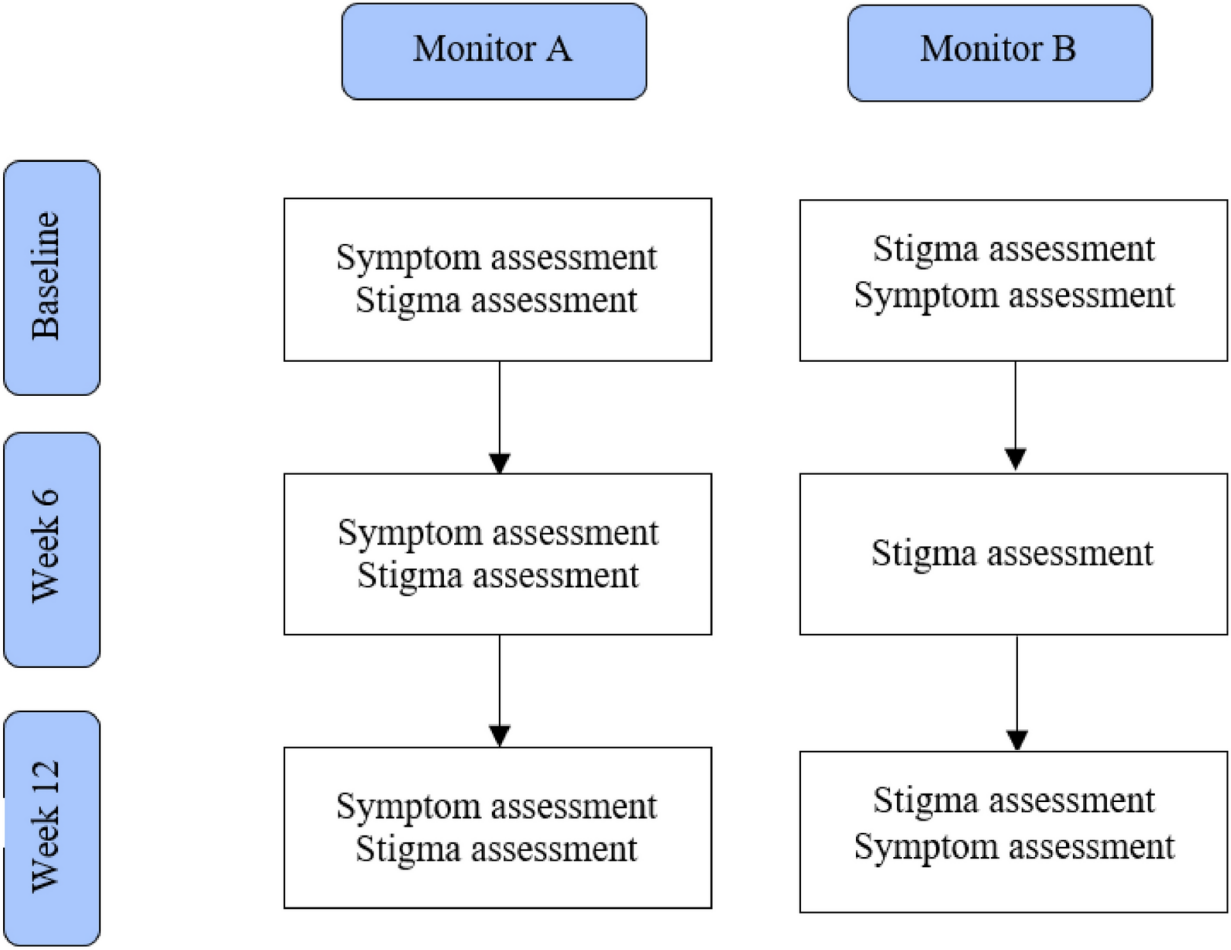
Flow of assessment for the Monitor A and Monitor B groups. The ordering of assessments differed between groups with the monitor A group presented with the symptom measure first and the monitor B group presented with the stigma assessment first. The monitor B group did not receive a symptom check at 6-week follow-up

The study design involved manipulating the ordering and frequency of assessments between groups. The monitor A group completed the symptom measure first, followed by the stigma assessment at each timepoint, whilst the monitor B group completed the stigma assessment at all timepoints, and completed the symptom measure *after* the stigma assessment only at baseline and at 12-week follow-up (see Fig. 2).

#### Randomisation

Each school was randomised including stratification procedures to ensure balance across conditions [61] on potentially important predictive factors: (1) socioeconomic status of the school using the Index of Community Socio-Educational Advantage level (ICSEA; < 1000 versus ≥ 1000), (2) location of the school (metropolitan vs rural/remote), and (3) gender mix of school (single vs mixed gender schools). Schools were randomised by an independent statistician on the basis of three variables collected in the demographics and screening questionnaires. Schools, parents, and children were not informed of their school’s allocation. Nine schools were randomised to either the monitor A or monitor B group, however three schools withdrew from the study following randomisation (see Fig. 1). Therefore, the final sample comprised of six participating schools allocated to the monitor A group (*k* = 4) or monitor B group (*k* = 2) and completed all three timepoints.

### Measures

#### Anxiety and depression symptoms

##### Revised Child Anxiety and Depression Scale (RCADS-C-25)

The child version of the 25-item Revised Child Anxiety and Depression Scale (RCADS-25; [62, 63]) was used to assess anxiety and depression in children in grades K to 6. The scales contain 15 items assessing anxiety symptoms and 10 items assessing major depression, rated on a 4-point Likert scale ranging from *Never* (0) to *Always* (3). Higher scores indicate higher levels of symptomology. Due to a lack of Australian normative data, the raw scores of the RCADS-C-25 were converted into t-scores using age and gendered normative data from the Netherlands [64]. The conversion formulae differed depending on their gender and the target responding grade (3–4 or 5–6). Upon receiving feedback from participants after baseline regarding lack of gender inclusivity, some scales were modified to replace gender binaries with gender-neutral terminology (e.g., “he/she” with “they”) (see Appendix A for modifications). As there are currently no normative data available for non-binary children, composite t-score formulas were created using the average of population means and standard deviations for boys and girls of each year group. T-scores of 65 and above for both anxiety and depression subscales were considered elevated, as did a 1SD significant increase in total t-scores across consecutive timepoints.

The RCADS-C-25 has demonstrated adequate internal consistency in community and clinical samples (Cronbach *α*’s = 0.70–0.82; [64]). The anxiety scale shows good validity and internal consistency (α = 0.82) with test–retest reliability (Intraclass correlation co-efficient [ICC] = 0.73) and criterion validity considered acceptable (Area Under the Curve [AUC] = 0.79), while the depression scale has acceptable test–retest reliability (ICC = 0.70) [64]. For the present study, the anxiety scale (Cronbach *α*’s = 0.87–0.89) and depression scale (Cronbach *α*’s = 0.83–0.88) demonstrated good internal consistency across all timepoints. Whilst the internal consistency for the total RCADS-C score was excellent (Cronbach *α*’s = 0.91–0.93).

#### Child report stigma

##### The Paediatric Self-Stigmatization Scale (PaedS)

The PaedS [16] is a self-report instrument used to measure different types of stigma and related experiences for children. It comprises four subscales which measure *Societal Devaluation* (14 items), *Personal Rejection* (5 items), *Self-Stigma* (5 items), and *Secrecy* (7 items). The *Societal Devaluation* subscale assesses a child’s perception of social stereotyping and discrimination in relation to trust issues, rejection by other children, and schooling. The *Self-Stigma* subscale measures feeling embarrassed about mental ill-health, feeling different from others, and having a sense of making others uncomfortable by one’s presence. The *Personal Rejection* subscale assesses feelings of isolation and rejection from peers due to mental ill-health. Whilst the *Secrecy* subscale examines how comfortable a child may feel about telling others about their mental health and receiving mental health treatment.

Items are rated on a 4-point Likert scale, except for the *Personal Rejection* subscale, which uses a binary “Yes/No” response. Four separate subscales scores were calculated by standardising the scores and then computing their average sum. An *Overall stigma* score, which assigns equal weight to each subscale, was also calculated by averaging the total scores of each of all four subscales. Higher scores on the subscales and overall stigma score indicates greater stigmatisation.

The PaedS was originally designed for clinical samples. Following participant feedback at baseline, the questionnaire was modified for the present study at the 6-week and 12-week follow up assessment timepoints to ensure relevance in settings where not all respondents were in receipt of treatment. The PaedS was modified by including a mental health screening item (at the 6-week and 12-week follow-up) asking participants whether they were currently receiving mental health treatment. Based on their response (i.e., “Yes”, “No”, “Prefer not to say”), some items were slightly reworded to ensure their relevance (see Appendix A for modified versions). For example, one item was adjusted to either “Are people rude to *you* because you feel really sad or worried?” or “Are people rude to *children* who feel really sad or worried?” to best align with the respondent. The PaedS was initially adapted from an adolescent version and validated for suitability in a child population aged 8 to 12 years, demonstrating good internal consistency across all subscales (Cronbach’s *α* = 0.72 to 0.86) [16]. The modified PaedS demonstrated good to excellent internal consistency for the *Societal devaluation* subscale (Cronbach *α* = 0.87 to 0.91); adequate to good internal consistency for the *Personal rejection* subscale (Cronbach *α* = 0.74 to 0.87); good to excellent for the *Self-stigma* subscale (Cronbach *α* = 0.88 to 0.91); and adequate for the *Secrecy* subscale (Cronbach *α* = 0.70 to 0.78). *Overall stigma scores* showed good to excellent internal consistency at all three timepoints (Cronbach *α* = 0.81 to 0.90).

### Procedure

#### Recruitment

Australian primary schools were recruited via social media advertisements, word-of-mouth, e-mail, and phone contact. The study was advertised “to test a new online mental health screening tool developed for primary school children and its impact on mental health stigma.” Information sheets describing the study were sent directly to school administrators who could register their interest via the study website or by contacting the research team. Online advertisements directed parents to the study website where they could register their interest. The research team contacted those who registered interest to provide further information about the study.

When a school registered interest, principals were directed to complete a brief background questionnaire to assess their school’s eligibility. If eligible, schools were notified via telephone by the research team. If ineligible, the respondent was directed to a webpage indicating that their school was not currently eligible for participation. Participating schools were asked to email the provided information about the study to parents including a video description, an information sheet, and an opt-out consent form. In addition, parents were invited to attend an online information session to learn more about the study details.

As an incentive for participation, schools were provided with a summary report on their school’s mental health results upon study completion. This report presented anonymous, aggregated data of the students’ anxiety and depression symptoms across school grades. Schools were encouraged to utilise this report to inform future wellbeing initiatives and resources within the school. Parents of participating students were informed that the school would receive a report based on the school’s mental health results. Further, as compensation for their time, all parents of participating children were entered into a prize draw to win one of two $250 gift vouchers, which was drawn at the completion of the trial.

#### Screening and consent

An opt-out consent process was used for children of participating schools. Researchers provided consenting schools with advertising material and study information to distribute to parents of their students. Parents were given a 2-week period to either consent to participate themselves, opt-out their child from participating, or both. Where parents did not opt-out their child, children were invited to participate in the research at school as part of a class activity. To obtain child consent, teachers distributed the survey link to children in-class and read aloud a script with information about the study. Upon clicking on the link, children were asked “*Are you happy to answer these questions about your mental health? If you start and decide to stop that is OK.*” If children consented, they proceeded to the questionnaires. If children did not consent, the teacher would arrange for them to participate in an alternate activity. Parents were given the option to withdraw their child from the study at any time.

After providing consent, participants completed a demographics questionnaire, which included questions about the child and parents’ age, gender identity, school, ethnicity, parent occupation, geographic area, and any intellectual disability diagnoses. The ethnicity question was broadly based on the Australian Standard Classification of Cultural and Ethnic Groups (Australian Bureau of Statistics), with participants given the choice to identify with the given categories or select ‘Other’ and provide free text. Key demographic information (i.e., school, age, and gender) was collected across timepoints and used to match participant responses. Additionally, participants created unique codes at each timepoint to ensure longitudinal responses were matched across assessments.

#### Data collection

Data collection was hosted online such that parents could access questionnaires remotely on their own device and children could complete them in the classroom on a device provided by their school. Data collection was conducted during a period post the COVID-19 pandemic when children were physically back in school settings. Schools were asked to allocate 15 min of in-class time for students to participate in the study at each timepoint. Participating children completed the questions independently and were informed that they could stop answering at any time and ask for teacher assistance if required. Responses to all items were optional to ensure a comfortable environment for children and to prevent any sense of coercion.

Participating parents were emailed links to their questionnaires to complete at each timepoint. A maximum of two reminders were sent via email at each instance to complete either their screening, baseline, 6-week follow-up, and 12-week follow-up questionnaires. Participants were withdrawn from the study if they did not complete the baseline assessment within 2 weeks of starting; or did not complete their follow-up surveys within 2 weeks of the scheduled dates. This was to ensure that the assessments accurately recorded a time-limited snapshot of the participant’s outcomes.

#### Statistical analysis

Statistical analyses were conducted using IBM SPSS Statistics software (Version 28.0). For the preliminary analyses, participant codes were matched across timepoints. For codes that did not match (e.g., typing errors), at least 3 identical demographic responses (i.e., school, grade, and gender) were required to confirm a matched participant. Severity analyses were conducted at the 6-week and 12-week timepoints to assess for any significant increases in anxiety and/or depression symptoms. Parents of children showing elevated scores were contacted by the research team and provided with the option to withdraw their child from the study. Fifty participants had elevated symptoms, however there were no withdrawals following these elevated scores.

For the primary analyses, a mixed-model for repeated measures (MMRM) was conducted to investigate between group differences (monitor A vs. monitor B) in *Overall stigma* (PaedS) over time (baseline, 6-week follow-up, 12-week follow-up), with school included as a random effect to accommodate clustering effects. To investigate within group differences in stigma over time, two separate MMRM analyses were conducted for the monitor A group and the monitor B group. Time was treated as a within-groups factor (baseline, 6-week follow-up, 12-week follow-up) with effects attributable to schools included as a random intercept.

Further secondary analyses were conducted with the stigma (PaedS) subscales; (i) *Societal Devaluation*, (ii) *Personal Rejection*, (iii) *Self-Stigma*, and (iv) *Secrecy* to examine any between group differences (monitor A vs. monitor B) across the various types of stigma. In addition, exploratory analyses were conducted with *Anxiety* and *Depression* (RCADS-C) as the outcome measures to investigate between group differences (monitor A vs. monitor B) in psychopathology at the 12-week follow-up compared to baseline. Separate MMRM analyses were run for the four stigma subscales, anxiety, and depression.

The MMRM approach handles missing data by including all available data from each subject into the analysis and assumes missing data are missing at random. Restricted maximum likelihood (REML) estimation was used in the analyses. An unstructured covariance matrix accommodated within-participant dependency, and degrees of freedom were estimated using the Kenward–Roger method. Between-groups effect sizes were calculated as the modelled standardized mean difference on each occasion of measurement. The primary analyses were two tailed, with statistical significance set at *p* < 0.05. For the exploratory analyses the alpha level was adjusted for multiple comparisons (*p* = 0.5/6). Effect sizes were calculated to determine the size of the within group changes between baseline, 6-week follow-up, and 12-week follow-up of the intervention for each outcome measure.

## Results

### Preliminary analyses

#### Sample characteristics

No significant differences were found between groups on individual demographics and any school-level differences are captured by the school random effect in the MMRM analysis.

### Primary outcome

#### Stigma

##### Overall stigma

**Fig. 3:**
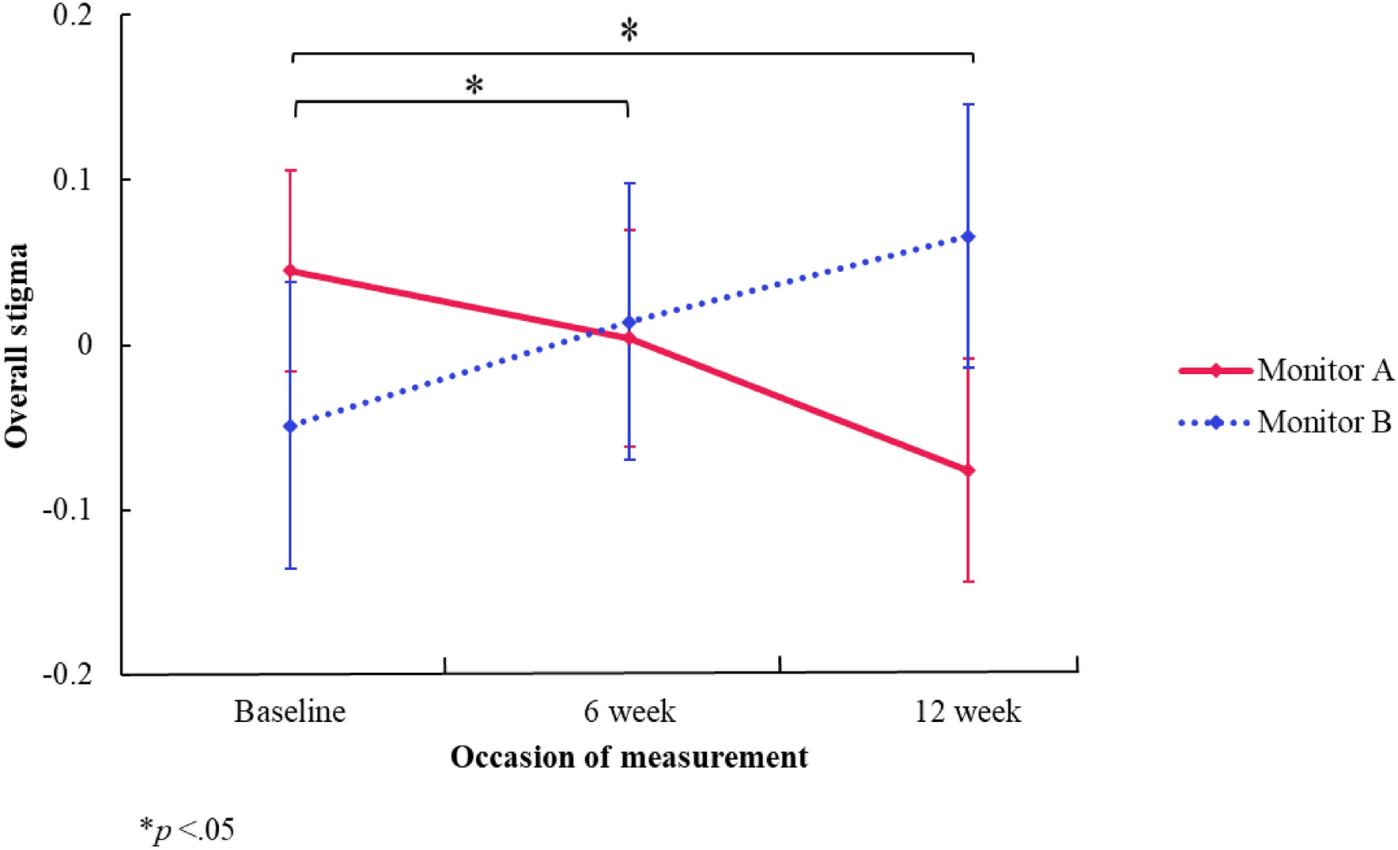
Overall stigma (PaedS) estimated marginal means by group and time

There was no significant *group* difference in overall stigma (*F*[1, 3.039] = 0.094, *p* = 0.277, 95% CI [− 0.131, 0.319]). There was a significant *time* effect showing a decrease in overall stigma at the 12-week follow-up (*M* = − 0.005) compared to baseline (*M* = − 0.002) (*F*[2, 398.515] = 0.115, *p* = 0.010, 95% CI [0.028, 0.202]), but no significant difference in overall stigma at the 6-week follow-up compared to baseline (*F*[1, 505.403] = 0.063, *p* = 0.082, 95% CI [− 0.008, 0.134]). There was a significant interaction effect (*group *×* time*), showing a difference in change in overall stigma between the groups at baseline and the 6-week follow-up (*F*[2, 527.045] = − 0.104, *p* = 0.027, 95% CI [− 0.197, − 0.012]), and baseline and the 12-week follow-up (*F*[2, 403.060] = − 0.237, *p* = < 0.001, 95% CI [− 0.349, − 0.124]). That is, the decrease in overall stigma in the monitor A group, at the 6-week follow-up (*M* = 0.004) compared to baseline (*M* = 0.045), was significantly different to the increase in overall stigma in the monitor B group, at the 6-week follow-up (*M* = 0.014) compared to baseline (*M* = − 0.049). Similarly, the decrease in overall stigma in the monitor A group, at the 12-week follow-up (*M* = − 0.076) compared to baseline (*M* = 0.045), was significantly different to the increase in overall stigma in the monitor B group, at the 12-week follow-up (*M* = 0.066) compared to baseline (*M* = − 0.049). See Fig. 3 for between group interaction effects.

### Secondary outcomes

#### Societal devaluation

**Fig. 4:**
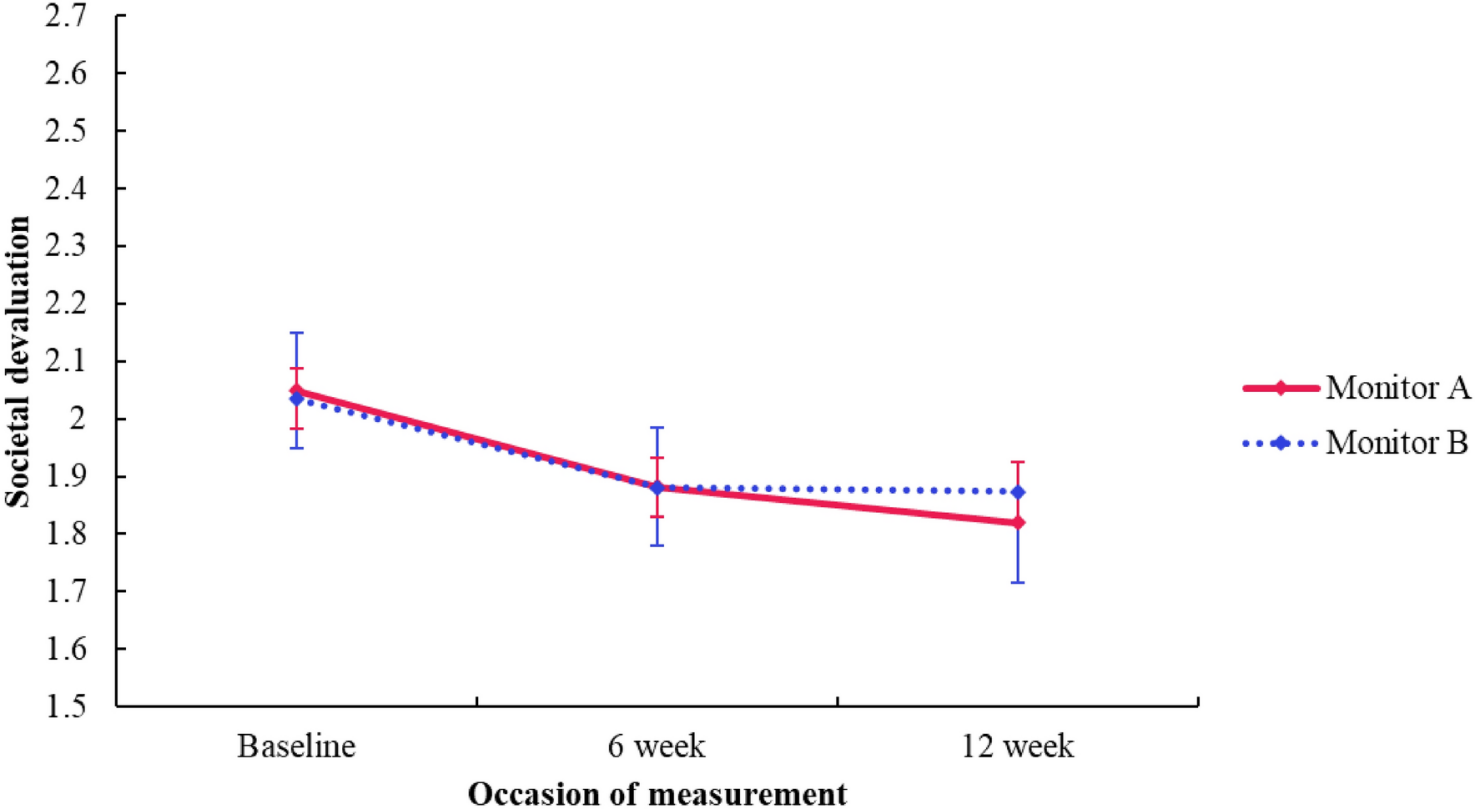
Societal devaluation subscale (PaedS) estimated marginal means by group and time

There was no significant *group* difference in concerns around societal devaluation (*F*[1, 3.247] = 0.014, *p* = 0.937, 95% CI [− 0.499, 0.528]). There was a significant *time* effect showing a decrease in concerns around societal devaluation from baseline (*M* = 2.04) compared to the 6-week follow-up (*M* = 1.88) (*F*[2, 496.880] = − 0.154, *p* < 0.001, 95% CI [− 0.216, − 0.092]), and baseline compared to the 12-week follow-up (*M* = 1.85) (*F*[2, 411.782] = − 0.162, *p* < 0.001, 95% CI [− 0.243, − 0.080]). These outcomes remain significant after adjusting for multiple comparisons. However, there was no significant interaction effect (*group x time*), that is, no difference in change in concerns around *Societal Devaluation* between the monitor A and monitor B groups at baseline and the 6-week follow-up (*F*[2, 514.306] = − 0.013, *p* = 0.751, 95% CI [− 0.094, 0.068]) or baseline and the 12-week follow-up (*F*[2, 412.769] = − 0.068, *p* = 0.203, 95% CI [− 0.172, 0.037]). See Fig. 4.

#### Personal rejection

**Fig. 5:**
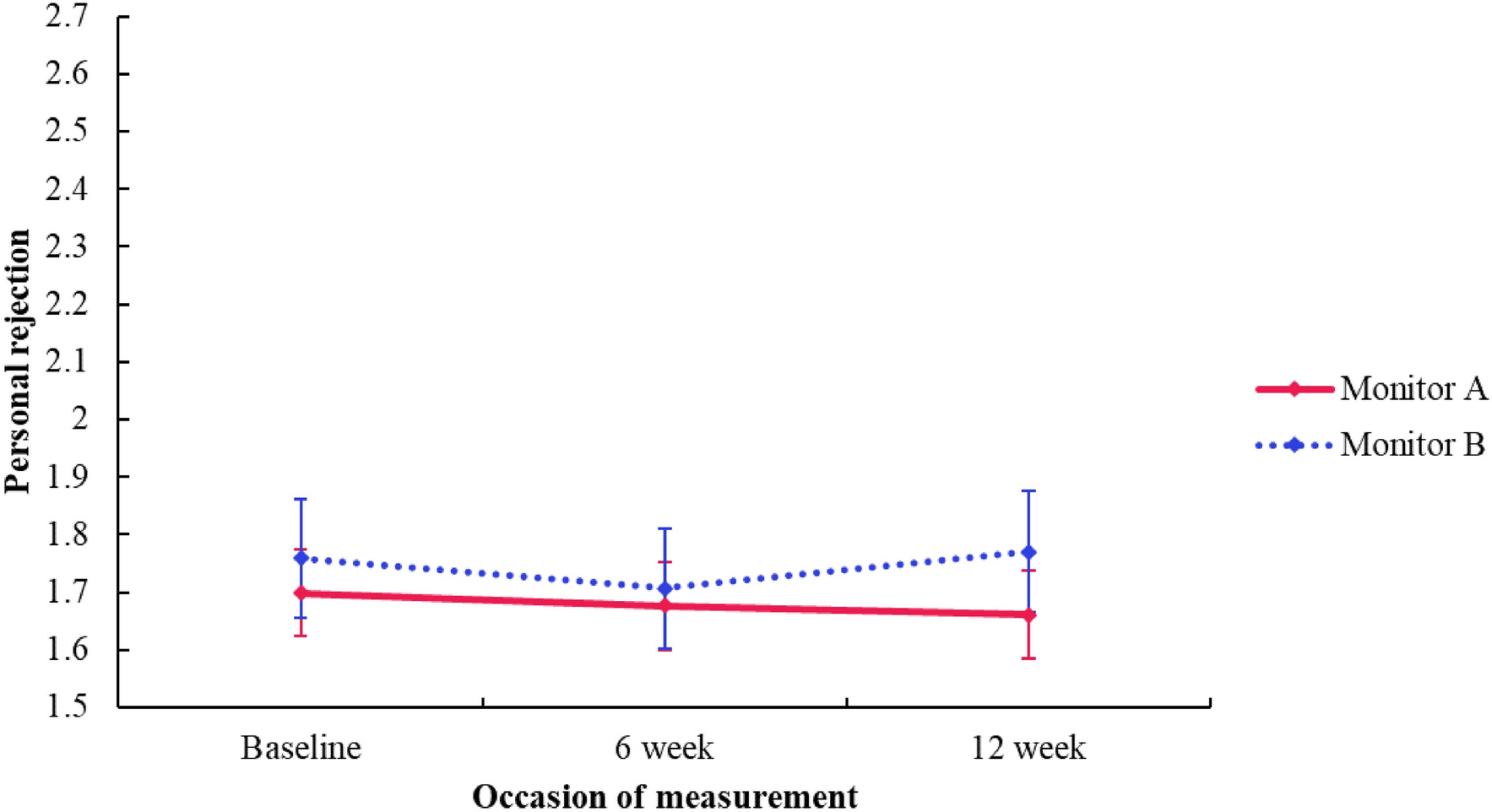
Personal rejection subscale (PaedS) estimated marginal means by group and time

There was no significant *group* difference in concerns around *personal rejection* (*F*[1, 3.180] = − 0.061, *p* = 0.662, 95% CI [− 0.453, 0.331]). There was a significant *time* effect showing a decrease in concerns around personal rejection from baseline (*M* = 1.729) compared to the 6-week follow-up (*M* = 1.691) (*F*[2, 513.279] = − 0.053, *p* = 0.034, 95% CI [− 0.102, − 0.004]), however it is acknowledged that this outcome would not remain significant if adjusted for multiple comparisons. There was no significant *time* effect of a difference in concerns around personal rejection from baseline compared to the 12-week follow-up (*F*[2, 404.067] = 0.011, *p* = 0.723, 95% CI [− 0.049, 0.070]). Further, there was no significant interaction effect (*group *×* time*), showing no difference in change in concerns around personal rejection between the monitor A and monitor B group at baseline and 6-week follow-up (*F*[2, 532.337] = 0.031, *p* = 0.340) or baseline and 12-week follow-up (*F*[2, 406.574] = − 0.048, *p* = 0.215, 95% CI [− 0.123, 0.028]). See Fig. 5.

#### Self-stigma

**Fig. 6:**
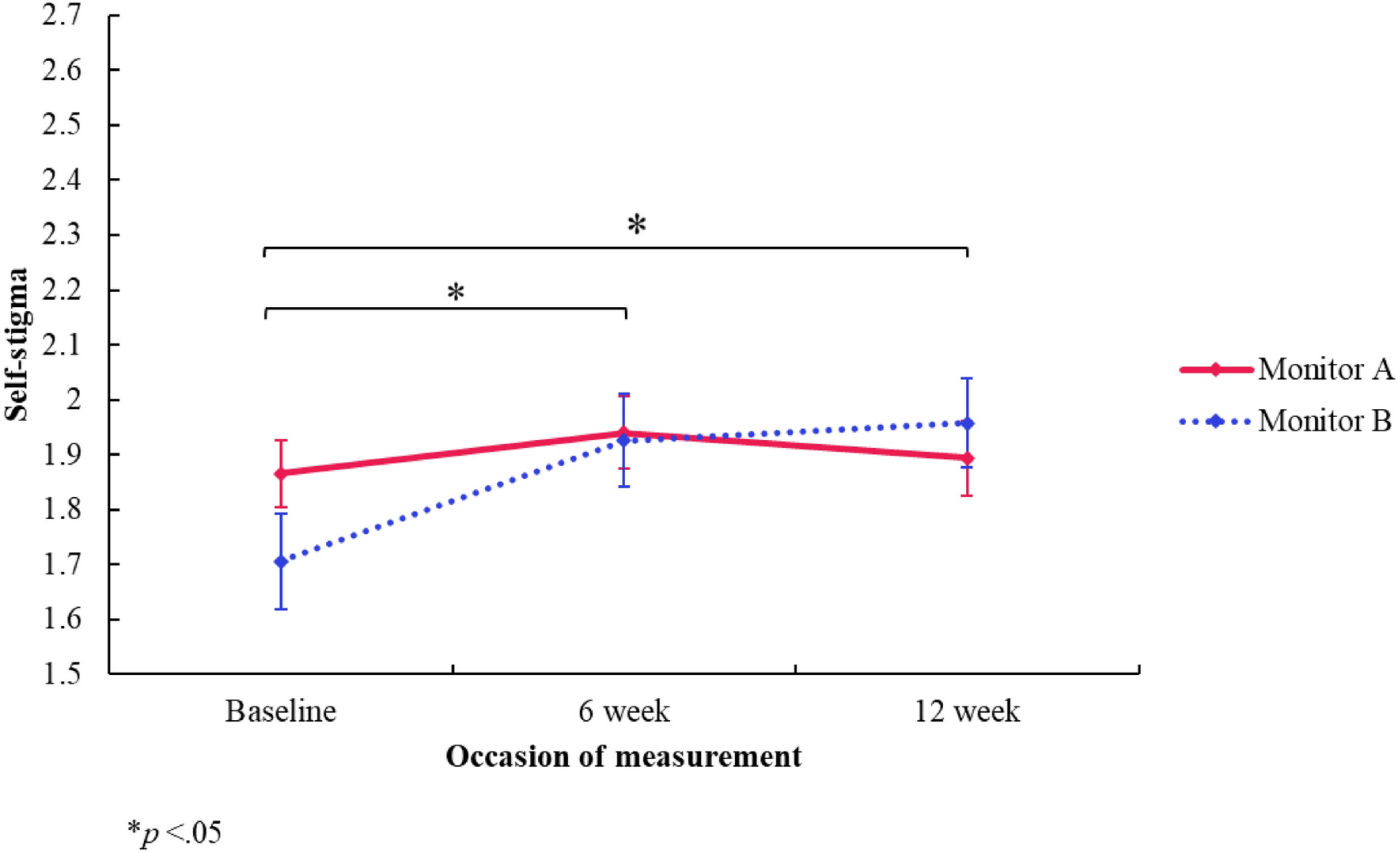
Self-stigma subscale (PaedS) estimated marginal means by group and time

There was no significant *group* difference in concerns around self-stigma (*F*[1, 2.014] = 0.160, *p* = 0.176, 95% CI [− 0.174, 0.493]). However, there was a significant *time* effect showing an increase in concerns around self-stigma from baseline (*M* = 1.79) compared to the 6-week follow-up (*M* = 1.93) (*F*[2, 512.800] = 0.220, *p* < 0.001, 95% CI [0.120, 0.319]), and baseline compared to the 12-week follow-up (*M* = 1.93) (*F*[2, 413.355] = 0.251, *p* < 0.001, 95% CI [0.128, 0.375]). These outcomes remain significant after adjusting for multiple comparisons. There was a significant interaction effect (*group x time*), showing a difference in change of concerns around self-stigma between the monitor A and monitor B group at baseline and the 6-week follow-up (*F*[2, 533.481] = − 0.146, *p* = 0.027, 95% CI [− 0.276, − 0.017]), and baseline and the 12-week follow-up (*F*[2, 416.846] = − 0.223, *p* = 0.006, 95% CI [− 0.380, − 0.066]) (see Fig. 6). That is, the increase in concerns around self-stigma in the monitor A group, at the 6-week follow-up (*M* = 1.94) compared to baseline (*M* = 1.87), was significantly different to the increase in concerns around self-stigma in the monitor B group, at the 6-week follow-up (*M* = 1.93) compared to baseline (*M* = 1.71). Similarly, the increase in concerns around self-stigma in the monitor A group, at the 12-week follow-up (*M* = 1.89) compared to baseline (*M* = 1.87), was significantly different to the increase in concerns around self-stigma in the monitor B group, at the 12-week follow-up (*M* = 1.96) compared to baseline (*M* = 1.71). However, if adjusted for multiple comparisons, these interaction effects would not remain significant and therefore should be interpreted with caution.

#### Secrecy

**Fig. 7:**
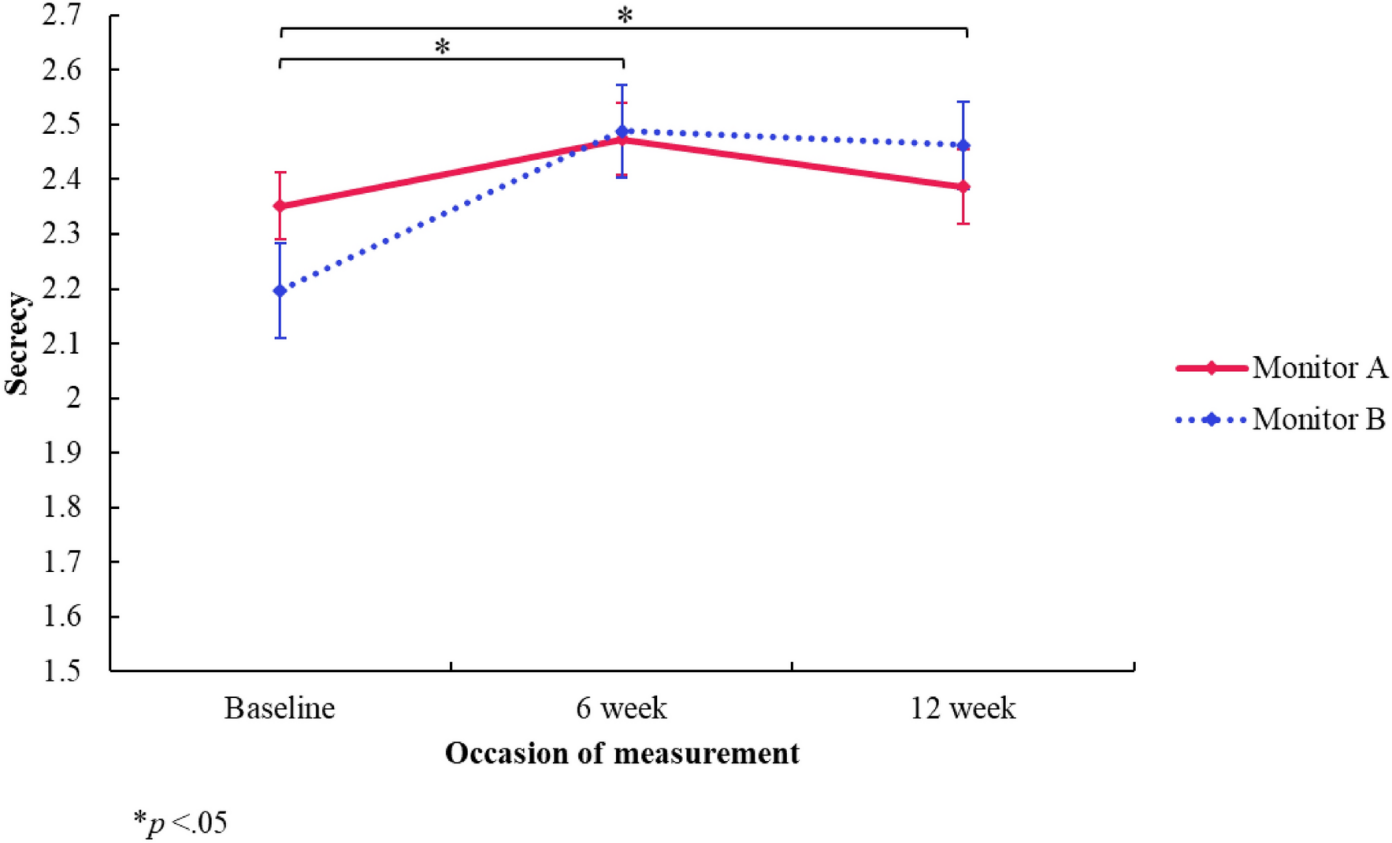
Secrecy subscale (PaedS) estimated marginal means by group and time

There was no significant *group* difference in concerns around secrecy (*F*[1, 2.323] = 0.154, *p* = 0.249, 95% CI [− 0.227, 0.535]). However, there was a significant *time* effect showing an increase in concerns around secrecy from baseline (*M* = 2.27) compared to the 6-week follow-up (*M* = 2.48) (*F*[2, 573.264] = 0.291, *p* < 0.001, 95% CI [0.192, 0.389]), and baseline compared to the 12-week follow-up (*M* = 2.42) (*F*[2, 441.497] = 0.266, *p* < 0.001, 95% CI [0.156, 0.377]). These outcomes would remain significant if adjusted for multiple comparisons. There was also a significant interaction effect (*group *×* time*), showing a difference in change of concerns around secrecy between the groups at baseline and the 6-week follow-up (*F*[2, 562.606] = − 0.168, *p* = 0.010, 95% CI [− 0.297, − 0.040]), and baseline and the 12-week follow-up (*F*[2, 448.085] = − 0.231, *p* = 0.001, 95% CI [− 0.372, − 0.090]) (see Fig. 7). That is, the increase in concerns around secrecy in the monitor A group, at the 6-week follow-up (*M* = 2.473) compared to baseline (*M* = 2.351), was significantly different to the increase in concerns around secrecy in the monitor B group, at the 6-week follow-up (*M* = 2.49) compared to baseline (*M* = 2.20). Similarly, the increase in concerns around secrecy in the monitor A group, at the 12-week follow-up (*M* = 2.39) compared to baseline (*M* = 2.35), was significantly different to the increase in concerns around secrecy in the monitor B group, at the 12-week follow-up (*M* = 2.46) compared to baseline (*M* = 2.20) (see Fig. 7). However, it is acknowledged that one significant interaction effect (6-week follow up compared to baseline) would not remain significant if adjusted for multiple comparisons, and therefore should be interpreted with caution.

#### Anxiety

**Fig. 8:**
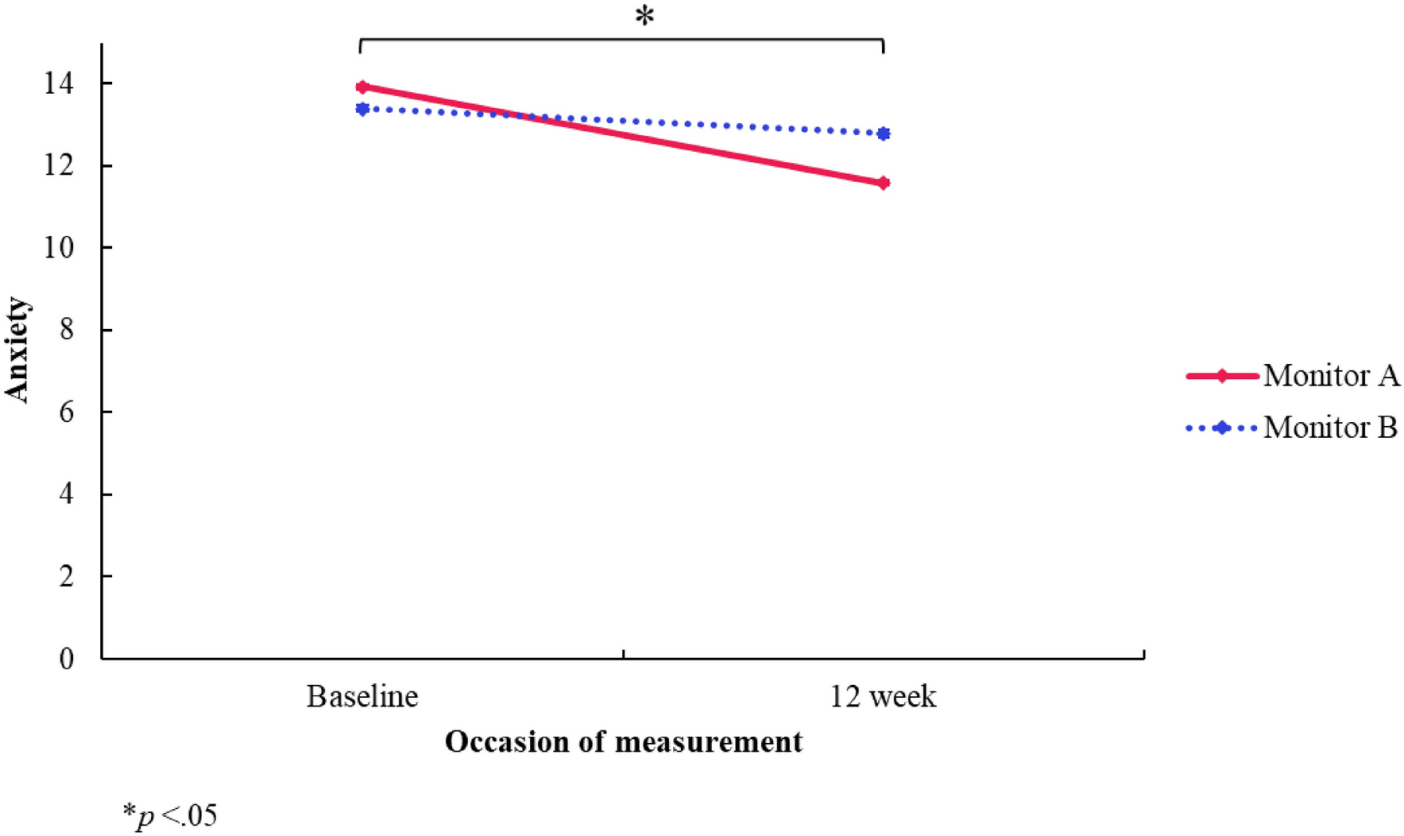
Anxiety subscale (RCADS-C) estimated marginal means by group and time

There was no significant *group* difference in anxiety (*F*[1, 3.399] = 0.533, *p* = 0.660, 95% CI [− 2.678, 3.834]). Further, there was no significant *time* difference in anxiety at 12-week follow-up compared to baseline (*F*[1, 319.032] = − 0.605, *p* = 0.272, 95% CI [− 1.689, 0.478]). However, there was a significant interaction *(group x time)* effect (*F*[1, 346.525] = − 1.735, *p* = 0.010, 95% CI [− 3.059, − 0.411), indicating that the decrease in anxiety in the monitor A group at 12-week follow-up (*M* = 11.59) from baseline (*M* = 13.93) was significantly different compared to the decrease in anxiety in the monitor B group at 12-week follow-up (*M* = 12.79) from baseline (*M* = 13.39) (see Fig. 8). This outcome remains significant if adjusted for multiple comparisons.

#### Depression

**Fig. 9:**
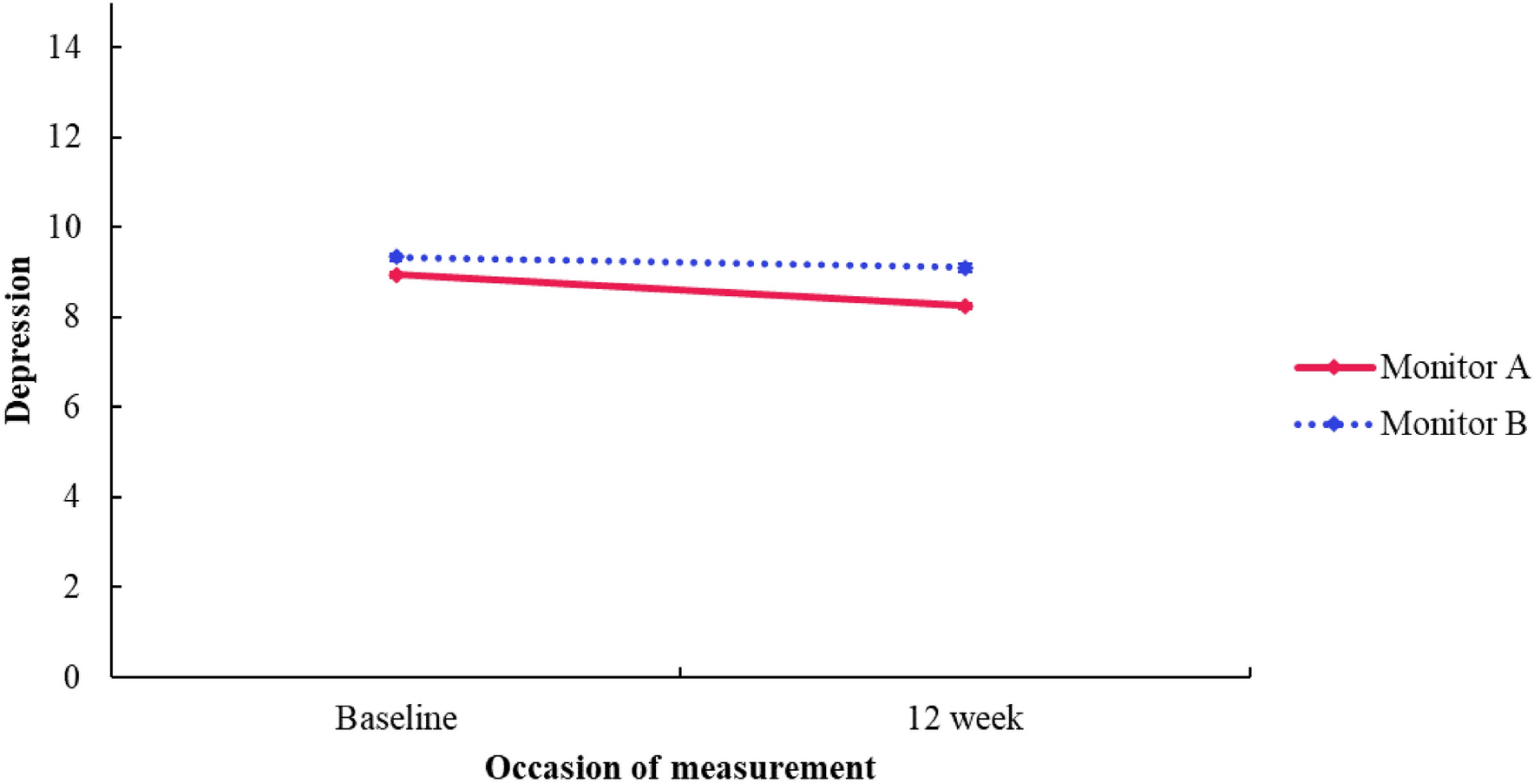
Depression subscale (RCADS-C) estimated marginal means by group and time

There was no significant *group* difference in depression (*F*[1, 1.705] = − 0.396, *p* = *0.5*57, 95% CI[− 3.200, 2.409). There also was no significant difference over *time* for depression at baseline and 12-week follow-up (*F*[1, 326.522] = − 0.234, *p* = *0.5*60, 95% CI [− 1.025, 0.556]). Further, there was no significant interaction effect (*F*[1, 348.801] = − 0.450, *p* = *0.3*63, 95% CI[− 1.422, 0.521]) (see Fig. 9).

## Discussion

The present study examined the potential harms and benefits associated with a universal mental health check in primary school children. Specifically, we investigated whether asking primary school children about their mental health in a school setting had an impact on mental health stigma and symptoms of anxiety and depression. Further exploration of the frequency and presentation order of the symptom check was conducted to assess its impact on children’s reports of mental health stigma. Overall, interaction effects were present for mental health stigma, indicating differing changes in mental health stigma between the two monitoring groups over time. Follow-up analyses of the stigma subscales showed significant interaction effects for self-stigma (feeling embarrassed about mental health) and secrecy (wanting to keep mental health problems a secret), but not for societal devaluation (public stigma relating to perceptions of social stereotypes) or personal rejection (feeling isolated and rejected from peers). The frequency and order in which questionnaires were presented to children had an impact on mental health stigma. Initially, children receiving the symptom check before the stigma measure reported heightened stigma, but over time, those receiving the symptom check more frequently experienced an overall stigma reduction (monitor A group), contrasting with an increase in overall stigma in those that received the symptom check less frequently and after the stigma measure (monitor B group). Finally, the study found interaction effects of a decrease in anxiety symptoms in those receiving the symptom check more frequently (monitor A), compared to those who received the symptom check less frequently and after the stigma measure (monitor B) group, over time. There was no significant between group differences in depression over time.

The present study findings suggest that asking primary school children about their mental health in a school-based setting had a significant impact on stigma over time. The results indicate that there is a possibility universal screening for mental health in primary school children may result in harm by worsening children’s attitudes and beliefs on mental health stigma, as well as views about their own and others mental health. The data indicate that whilst there was an overall reduction in mental health stigma in those that received the symptom check before the stigma measure and more frequently at all timepoints, there was an increase in mental health stigma in those that received the symptom check less frequently and after the stigma measure. These overall effects appear to be driven by the distinct pattern of results of the stigma subscales, as well as the effects observed in those that received the symptom check less frequently and after the stigma measure (monitor B group). Further inspection of the stigma subscales showed that the subscale measuring public stigma (i.e., societal devaluation) performed differently to the subscales measuring stigma related to the self (i.e., self-stigma, secrecy, and personal rejection). Whilst there were no significant differences between groups in the societal devaluation and personal rejection subscales, the self-stigma and secrecy subscales showed a similar pattern of results. The secrecy and self-stigma subscales demonstrated a worsening in stigma for those in the monitor B group compared to those in the monitor A group, suggesting that the sequence and frequency of mental health assessments affect children’s apprehension about feeling embarrassed regarding mental health issues and their reluctance to discuss their experiences with peers. Some studies have found no significant improvements in mental health stigma following a school-based intervention [38, 40, 42], and two studies showed an inconsistent pattern of results on stigma subscales depending on the measures used [39, 41]. Stigma can manifest in a variety of ways and the present study provides insights into the impact of a universal mental health check on different types of stigma in primary school children. This highlights the importance of taking into consideration the multidimensional nature of stigma when measuring the construct in order to capture and better understand the process of stigmatisation faced by young people [65].

In terms of the study design, the order and frequency of the assessments played an important role in the impact on stigma in children. The groups differed in terms of the presentation order and frequency of the stigma and mental health questionnaires. The findings suggest that asking children about stigma before their mental health (i.e., the monitor B group) may have the potential to significantly worsen overall stigma in primary school children over time. In contrast, the opposite trend of a reduction in overall stigma appeared when children were asked about their mental health first before stigma, and when more frequent mental health checks were present across all three timepoints (i.e., the monitor A group). Moreover, the pattern of results in the monitor A group showed that when children reported on their own mental health first before stigma, this initially led to higher ratings of stigma at baseline compared to the monitor B group, however over time at the 6-week and 12-week follow-ups, the monitor A group showed lower ratings of stigma long-term. In comparison, children in the monitor B group reported on stigma before completing questionnaires about their own mental health, and this mental health check was not present at the 6-week follow-up. Thus, the study design suggests that there may be long-term benefits for presenting questionnaires about mental health before stigma to children, and including more frequent mental health checks may help reduce stigma over time. Careful consideration of study design should be taken into account when developing universal checks as it may have important implications for the effectiveness and outcomes of school-based mental health interventions [29].

The findings also showed that for the monitor A group, repeated assessment of symptom checks was associated with a reduction of symptoms of anxiety over time, but not for depression. Perhaps the pattern of results may be explained by the large variability in the age of onset of anxiety disorders and depression in children and adolescents. A recent global meta-analysis showed that the age of onset of anxiety disorders (peak age of onset: 5.5 years; median age of onset: 17 years) was much younger compared to the age of onset of depression (peak age of onset 19.5 years; median age of onset: 30 years) based on parent retrospective reports [66]. Further, the proportion of individuals with onset of anxiety before the age of 14 was 38.1% compared to 3.1% for depression, suggesting that first symptoms of anxiety occurs much earlier than depression, which typically manifests in late childhood or adolescence [59, 66]. The current study was conducted with primary school aged children and in line with previous literature, showed reductions in anxiety symptoms, but not for depression. Exploring these findings in high school students may reveal stronger effects of reductions in symptoms of depression, however we weren’t able to find it in our current sample. Given that anxiety and depression are highly co-morbid, universal screening and early intervention approaches targeting primary school aged children before or during the onset of mental disorders may help improve mental health outcomes in the longer term for both anxiety and depression.

## Strengths and limitations

The findings of the present study must be interpreted against the strengths and limitations of this research. The study has many strengths. First, to our knowledge, this was the first cluster randomised controlled trial to investigate the impact of a universal mental health screening tool on mental health stigma in primary school children. Second, the study examined the impact of a universal mental health check on different types of mental health stigma, contributing to a better understanding of the multi-dimensional concept of stigma [29]. Another strength of the study is the unique study design which provided insights into the impact of delivering universal checks in primary school aged children on stigma and how and when asking children about their mental health can impact stigma. This has important implications for optimising the effectiveness of school-based mental health interventions. Finally, a strength of the study is the inclusion of multiple follow-up timepoints to assess stigma at 6-and 12-week follow-up. Most studies in the current literature do not include long-term follow-ups of mental health stigma assessments, with limited and mixed evidence on the long-term effectiveness of universal school-based mental health interventions [, 67].

There are also some limitations worth noting. First, there was a small number of participating schools in the study for a cluster randomized controlled trial, with an unbalanced number of schools in the monitor A (n = 4) and monitor B group (n = 2). Schools were initially randomised based on socioeconomic status of the school, location of the school (metropolitan vs rural/remote), and gender mix of school (single vs mixed gender schools). However, there were some schools that withdrew from the study due to reasons out of the researchers’ control (e.g., scheduling and administrative issues, concerns regarding the mental health content). In addition, recruiting schools was challenging due to the study being conducted after the COVID-19 pandemic, during a period when there were increased pressures on schools and a greater workload for teaching staff, as children were returning to the classroom following extended periods of lockdowns and uncertainty. A second limitation of the current study is sampling bias may limit the generalisability of the findings, as participating schools were predominantly Independent schools from New South Wales. Whilst efforts were made to include a representative sample of schools, restrictions due to COVID-19 during the time of recruitment and different ethical procedures resulted in limited opportunities for more government schools to participate in the study. Nevertheless, despite the limitations, the present study provides important implications for disseminating school-based universal mental health checks in primary schools and provides a solid foundation for future studies, with critical insights for children, parents and stakeholders in the education, public health, and policy sectors.

## Future research

The results of the present study suggest that the possibility of harm cannot be excluded from introducing universal mental health checks in primary schools. These findings need to be replicated. They also highlight the need to fully understand the impact of asking children about their mental health within the school context, when they are surrounded by their peers and teachers (who represent the feared stimuli for many children with anxiety and depression), as not to worsen stigma. Further studies in this area should focus on resolving methodological issues on how outcomes are assessed, which will better inform the design of the intervention and improve delivery and implementation [29]. For instance, future studies could extend the study design by including the symptom check at the 6-week follow-up for both groups, as well as not including any mental health check at any time point. These variations in study design would help us to further understand the impact of universal school-based mental health checks on stigma and guide effective administration. Future studies should aim to include a more diverse sample of schools (e.g., different types of schools, sizes, locations, socio-economic backgrounds), which would lead to a more representative sample and generalisable results.

The results of the present study relied on primary school children’s self-report on measures of stigma and mental health. This has implications for the high degree of variability in self-report, insights into their mental health, and reading ability at this age (grades 3 to 6). Whilst these factors may influence the results, children completed the study in the classroom where they were encouraged to ask teachers for help if they were unsure about the questions. For future studies, using either parent or teacher reports for primary school aged children, or consolidating parent, teacher, and child reports, may provide a more accurate measurement of mental health and stigma. Parents were included in the present study, however as this was primarily a school-based study, there was a poor response rate and low engagement from parents perhaps due to recruitment focus primarily occurring via schools, or lack of time commitment from parents to participate in the study. Future studies should prioritise improving parent engagement to include parent data and capture measurement from different perspectives, particularly for children in Grades K to 2. Incorporating longer-term follow-ups to assess the effectiveness of universal mental health checks and assess their sustainability over time would also be beneficial. Further, including gender neutral pronouns and ensuring questionnaires are gender inclusive is a limitation that needs to be addressed in the wider field. Based on feedback from participants in the current study, questionnaires were modified to be more gender inclusive for diverse identities, and future studies should ensure all questionnaires are appropriate and inclusive for participants, in particular studies concerning mental health stigma.

Finally, a consideration for future research is to focus on developing clear and standardised definitions of mental health stigma to incorporate the multifaceted dimensions of this construct [14, 16]. Studies often show mixed results in terms of the effectiveness of universal checks and impact on stigma, which may be due to the different way stigma is conceptualised, measured, and operationalised [30–37]. Clearly defining the construct to include what constitutes mental health stigma and using appropriate measurement tools will help future studies provide a clearer picture of stigma in children.

In conclusion, the present study suggests although we observed an overall reduction in stigma following repeated assessments of anxiety and depression, caution is needed when asking primary school children questions about stigma and mental health in universal school-based mental health screening. There is the possibility that measurement may worsen children’s views about their own and others’ mental health. In particular, potential screening harms, such as exacerbating self-stigma and secrecy aspects of stigma, warrant consideration. The frequency and presentation order of mental health checks impact children’s reports of mental health stigma, emphasising the importance of screening context, study design, and implementation. Addressing stigma related barriers is crucial for enhancing mental health care access for children in schools and improving outcomes.

## Supplementary Information

Supplementary Material 1.

## Data Availability

The data collected and analysed in the current trial is not currently available to researchers outside of the approved team due to constraints placed on the project by the various ethics bodies. Additional related project documents are currently available from the Australian and New Zealand Clinical Trials Register (ACTRN12622001114730).
