# Supplementary material for: The impact of universal mental health screening on stigma in primary schools
Source: Child Adolesc Psychiatry Ment Health. 2025 Jan 29;19:5. doi: 10.1186/s13034-024-00854-5 (PMC11780817; doi:10.1186/s13034-024-00854-5)
Supplement: Supplementary file 1 — Supplementary Material 1. [file 13034_2024_854_MOESM1_ESM.docx]

**Supplementary Material**

**Appendix A**

Based on participant feedback at baseline, modifications were made to questionnaires for the current study to ensure that items were relevant for a non-clinical sample of children. In addition, questionnaire items were modified due to lack of gender inclusivity and replacing gender binaries with gender-neutral terminology (e.g., “he/she” with “they”).

Only the modified questionnaires relevant for the present study’s research questions are detailed below. The modified text is indicated in *italics*. All item numbers correspond to those in the original questionnaire.

**The Paediatric Self Stigma Scale (PaedS)**

| **Item** | **Original item** | **Modified item** |
| --- | --- | --- |
| N/A | N/A | *The following instructions were added:*  *Have you ever heard the words 'mental health' before? Maybe you have heard them before. Maybe these are new words. We want to ask you what you think about mental health. Before we do, let's find out more about what these words mean. Let's think about health first. When you see the word health you probably think of your body. Being physically healthy means that your body is working the way it should and you don't have to struggle to do the things you need to do, like go to school or play. You don't have a cold or you are not sick. When we talk about mental health, it is about how healthy your mind or your feelings are. Some kids who struggle with their mental health, might feel really sad or really worried. They might cry or act really shy in front of other kids. They might see a doctor, or a counsellor or a psychologist to help them. This is called mental health treatment. For example, Billie struggles with feeling worried ALL the time. Most days they can't concentrate on their schoolwork. Lani feels sad a lot and feels like crying most of the time. She never feels like playing with other kids.*  *Read each question. Do you agree or disagree? Use the scale to help you answer.*  *Here is an example. Most children like ice cream. Do you agree or disagree?*  *1 = I disagree a lot, 2 = I disagree, 3 = I agree, 4 = I agree a lot*  *If you agree that most other kids like ice cream, you circle the number 4.*  *Now let’s try it with these. Read each one. Do you agree or disagree?*  *Use the scale to help you answer. 1 = I disagree a lot, 2 = I disagree, 3 = I agree, 4 = I agree a lot* |
| 1 | *Most* children my age *will* bully children if they know he/she is receiving mental health treatment. | Children my age bully kids *who are getting help for feeling really sad or worried*. |
| 2 | *Most* people believe that children with difficult feelings or behaviour are just as clever as other children. | People believe that children *who feel really sad or worried* are just as clever as other children. |
| 3 | *Most* children look down on other children receiving mental health treatment. | Children look down *or judge* other children *who are getting help for feeling really sad or worried*. |
| 4 | Most believe that a child with difficult feelings or behaviour is dangerous. | *People* believe that *children who feel really sad or worried* are dangerous. |
| 5 | Many are afraid of children who are getting mental health treatment. | *People* are afraid of children who are getting *help for feeling really sad or worried.* |
| 6 | People believe that children with difficult feelings or behaviour are to blame for their problems. | People believe that children *who feel really sad or worried* are to blame for their problems. |
| 7 | *Most* schools would worry about having children with difficult feelings or behaviour at their school. | Schools would worry about having children *who feel really sad or worried* at their school. |
| 8 | *Most* children would not want to play with somebody that has difficult feelings or behaviour. | Children *don’t* want to *play with somebody who* feels really sad or worried. |
| 9 | *Most* people believe that children with difficult feelings or behaviour cannot be trusted. | People believe that children *who feel really sad or worried* cannot be trusted. |
| 10 | *Most* people believe that children with difficult feelings or behaviour will never get better. | People believe that children *who feel really sad or worried* will never get better. |
| 11 | *Most* people believe that children with mental health problems cannot get good results in school. | People believe that *children who feel really sad or worried* cannot get good results in school. |
| 12 | *Most* people believe that children with difficult feelings or behaviour cannot take care of themselves. | People believe that children *who feel really sad or worried* cannot take care of themselves. |
| 13 | Teachers and other school staff give children with difficult feelings or behaviour a hard time. | Teachers and school staff *pick on* children *who feel really sad or worried.* |
| 14 | Most girls/boys will not date someone who has mental health issues. | Most *people* will not date someone who *feels really sad or worried* |

The following question was added to the Paediatric Self Stigma Scale (PaedS) at time points 2 and 3 (6-month follow-up and 12-month follow-up):

*Are you seeing a doctor or a counsellor to help you with feeling really sad or worried? Remember these answers are confidential, which means we won't share them with your teachers or anyone. YES/NO*

The below show different versions of the remaining PaedS items, tailored depending on their response this question.

| **Item** | **Original item** | **Answered yes to additional question** | **Answered no to additional question** |
| --- | --- | --- | --- |
| 15 | *Do you ever feel like* people are rude to you because of your difficult feelings or behaviour? | *Are people* rude to you because *you feel really sad or worried*? | Are people rude to *children* *who* feel really sad or worried? |
| 16 | Have people used the fact that you are receiving help to hurt your feelings? | Have *kids picked on you because you’re getting help (from a doctor or counsellor for feeling really sad or worried)?* | Do people pick on *kids who* get help (from a doctor or counsellor) for feeling really sad or worried? |
| 17 | Do *you ever feel like* people look down on you when they find out you are receiving help? | Do people *think badly of you for getting help (from a doctor or counsellor for feeling really sad or worried)?* | Do people think badly of *kids who* get help (from a doctor or counsellor) for feeling really sad or worried? |
| 18 | Have *you ever been* avoided *by* people because they knew you were getting treatment for difficult feelings or behaviour? | Have people avoided you *(don’t want to play with you)* because *you’re seeing a doctor or counsellor for feeling really sad or worried?* | *Do* people avoid *kids* (don’t want to play with them) because they’re getting help for feeling really sad or worried? |
| 19 | Did some friends reject you after they found out you were receiving help? | *Have* friends rejected you *(won’t play with you) because you’re seeing a doctor or counsellor for feeling really sad or worried?* | *Do kids* reject *(won’t play with) other kids* who are seeing a doctor or counsellor to help them with their feelings? |
| 20 | How often do you feel different from other children your age because you have difficult feelings or behaviour? | Do you feel different from other kids your age because you feel really sad or worried? | *If you felt really sad or worried, would you* feel different from other kids your age? |
| 21 | How often do you feel people may not like you if they know you have difficult feelings or behaviour? | Do you *think* people *won’t* like you *because you feel really sad or worried*? | *If you felt really sad or worried, would* people not like you? |
| 22 | *How often* do you feel people will not want to be friends with you if they know you have difficult feelings or behaviour? | Do you *think* people *don’t* want to be *your friend because you feel really sad or worried*? | *If you felt really sad or worried, would* people not want to be your friend? |
| 23 | *How often* do you worry that other people are uncomfortable with you because of your difficult feelings or behaviour? | Do you *think people feel* uncomfortable *(weird) around you because you feel really sad or worried*? | *If you felt really sad or worried, would* other people feel uncomfortable (weird) around you? |
| 24 | *How often* do you feel embarrassed about your difficult feelings or behaviour? | Do you feel embarrassed *because you feel really sad or worried*? | *If you felt really sad or worried, would* you feel embarrassed? |
| 25 | *There is no reason for a* person to hide the fact that he or she is receiving help for difficult feelings or behaviour. | People *shouldn’t* hide *that they are getting help (from a doctor or counsellor) because they feel sad or worried*. | People shouldn’t hide that they are getting help (from a doctor or counsellor) because they feel really sad or worried. |
| 26 | *I usually wait until I know a person really well before* I tell them I am receiving help for difficult feelings or behaviour. | I *only* tell *my best friend(s) that I’m getting help for feeling really sad or worried*. | *If I felt really sad or worried*, I’d only tell my best friend(s) *if I was* getting help. |
| 27 | When I meet people for the first time, I make a special effort to keep the fact that I am receiving help to myself. | *I don’t tell new people that I’m getting help for feeling really sad or worried.* | *If I felt really sad or worried,* I wouldn’t tell new people *if I was* getting help. |
| 28 | I *often* worry that someone will tell others about my difficult feelings or behaviour without my permission. | I worry that someone will tell others *that I feel really sad or worried behind my back.* | *If I felt really sad or worried*, I’d worry that kids would talk behind my back. |
| 29 | I *feel like* I need to hide *the fact* that I have difficult feelings or behaviour from children my age. | I need to hide that *I feel really sad or worried from other kids my age.* | *If I felt really sad or worried*, I’d need to hide it from other kids my age. |
| 30 | I *often feel the* need to hide the fact that I am receiving help. | I need to hide that *I’m getting help from a doctor or counsellor for my feelings.* | *If I was getting help* from a doctor or counsellor for my feelings, I’d hide it from other kids my age. |
| 31 | *If you are getting help with your difficult feelings or behaviour, the best thing to do is keep it to yourself.* | *I don’t want to tell anyone that I’m getting help from a doctor or counsellor for my feelings.* | *If I was getting help* from a doctor or counsellor for my feelings, I wouldn’t tell anyone. |

**The Revised Child Anxiety and Depression Scale – Child version (RCADS-C-25)**

Item 19 was modified for clarity due to feedback from participant’s misunderstanding the question.

| **Item** | **Original item** | **Modified item** |
| --- | --- | --- |
| 19 | I feel like I don’t want to move. | I feel *like I don’t want to get up and move or be active.* |
